# Mr. Shoolbred, on Vaccine Inoculation in Bengal

**Published:** 1805-04-01

**Authors:** John Ring

**Affiliations:** New Street, Hanover Square


### Mr. Shoolbred, on Vaccine Inoculation in Bengal.

( Continued from pp. 245—257. )

I shall prefix to the Analysis of Mr. Shoolbred's Report, a copy of a letter I received from him, together with the Report.

" SIR, *Calcutta, June* 21, 1804.

" HAVING observed that you have been at uncommon pains in collecting information from all quarters, on the subject of vaccine inoculation, and in tracing the history of its progress from the first promulgation of its discovery by the immortal Jenner up to the present day; I presume it will afford you some gratification to know what has been done in India, with a view to the preservation and dissemination of so great a blessing to mankind: I therefore beg leave to present you with a copy of the Report on the introduction of the vaccine disease into India, and its progress in Bengal, up to the conclusion of last year, which has just been published by order of this Government.

" You will observe, that this small work is to be considered as merely an official communication to the Bengal Medical Board, for the information of Government; not as a regular treatise on the vaccine disease, written with a view to its coming before the public. It is therefore entitled to much indulgence.

" You will also perceive, that I have taken the liberty of making use of some part of your valuable work, of which I have hitherto seen only the first volume; and that in such a way, as only to obtain a very hasty and cursory perusal of it; a circumstance necessary to be mentioned, lest I may have stated any thing which a more careful perusal might induce me to alter. I am impatient to see your your second volume, which I remark as being published, though it has not reached this country.

"The last communication I saw of your's was in the Medical and Physical Journal, for Dec. 1803; in which, you state your opinion, that the heat of the weather does not diminish the activity of the vaccine fluid, when immediately transferred from arm to arm. I was of the same opinion while the cold weather lasted; but when the thermometer rose to upwards of 90, I was convinced, by a very alarming number of failures, that the fact, however difficult to account for, was certainly otherwise.

"The infection was last year lost at one of the stations, from this cause. It is now extinct in two others, and has been in jeopardy in my own hands; on no other account which I can discover, but that the fluid at a very high temperature loses much of its virtue, even when used at the instant of its secretion. How this happens, while the disease, when it takes place, exhibits all its genuine characteristics, I am unable to explain.

"I have not observed any difference in the susceptibility of infection, depending on different shades of colour; nor does your observation on the rapid progress of the disease in negroes, appear to hold good in the black natives of Bengal. The course here is much the same as in Europe; with no difference between blacks and whites sufficient to attract observation.

"I am, &c.

"JOHN SHOOLBRED,

"Supt. General of Vaccination in Bengal."

Here it is necessary to remark, that Mr. Shoolbred notices one rule, on which I lay a particular stress respecting the practice in hot climates, but not another, which I consider equally indispensable, namely, that of taking the matter *at a very early period.*

He says, indeed, there is no material difference in the progress of the cow-pock in blacks and whites. On this point I am not competent to decide; my information was derived from the report of Dr. Dupuytren of Paris; who had much better opportunities of ascertaining the fact. We all know, however, that the progress of the disease is most rapid in hot weather; for which it no where appears, that Mr. Shoolbred has made any allowance, either in his inferences or his practice.

In the summer of 1800, I inoculated six children, with two punctures in each arm, from a child in Marshall Street, London

London Road; and out of the twenty-four punctures, only one succeeded. Yet subsequent inoculations proved, that neither of the subjects, on whom the operation failed, had previously had the small-pox.

At that time I followed the rule laid down by the latest writers; that of taking matter at any period when I could find it. In the present instance, the weather was extremely warm, the areola very vivid, the vesicle very large; I am, therefore, at present, not surprised at the result.

I am well convinced, from the concurrent testimony of the various authors who have given an account of the phenomena of vaccination in India, that the disease is more rapid there than in this northern climate. Yet Mr. Shoolbred's general rule was to take matter on the eighth day; a day when the matter is sometimes almost effete, in warm weather, even in this country, as I have repeatedly experienced.

But I am still more fully convinced of the rapidity of the vaccine pustule in India, from a representation of it accompanying the Report sent me by Mr. Shoolbred; in which the areola on the fourth day is as large as it is here on the tenth. As far as it is possible to draw a conclusion from this plate, the matter ought to be taken on the third day. This has been done, in cases of emergency, even in our cold climate, and with success; it is therefore worth attempting in India, where the lives of millions depend on perpetuating the practice.

But I have another, and a stronger reason, for supposing that the matter in India was not taken at a proper period; which is, that Dr. Marshall preserved the vaccine infection under a still greater degree of heat. In the Medical and Physical Journal for January, 1804, is an extract of a letter I received from him on that subject, in which he says, "In this month's Medical Journal, I observe a letter from Mr. Weston of Jamaica, to you, is inserted; in which he says, he is obliged to acknowledge the lamentable fact, that in a temperature of 90, the vaccine matter loses its activity.

"This to me appears a very extraordinary acknowledgment; for during the time that I was at Gibraltar, in Aug. 1800, the temperature was never so low as 90, and frequently at 96; during which time I never observed any particular difficulty in communicating infection, more than in England; nor can I conceive any probable cause for its failure in Jamaica."

Dr. Marshall adds, "From my original memorandum, now now before me, I find your observation upon the rapid progress of the disease in negroes just; since the arm of this man yielded matter for inoculation on the third day." Here was no motive for taking matter at a very early period; on the contrary, Dr. Marshall wished to protract his inoculations, in order to convey recent matter to Gibraltar. It is therefore reasonable to suppose, that there was a distinct vesicle, fit for the purpose of inoculation. A case mentioned by Dr. Coxe rather confirms the idea of the disease being more rapid in the Negro. That it is more rapid in a warm temperature is certain; and, I trust, in future, this circumstance will be particularly remembered in practice.

Mr. Shoolbred commences his Report with a concise view of the introduction of the disease into India, and the means employed to ensure its permanent establishment in that quarter of the world, previous to his appointment. The office of Superintendant General of Vaccination was first held by Mr. Russel, who discharged the duties of that office in an exemplary manner, till he was obliged to leave India on account of ill health. All the European children in Calcutta and its neighbourhood were speedily vaccinated; and the blessings of the practice were widely diffused.

Previously to Mr. Shoolbred's succession to that important office, he had kept up a series of successive vaccinations at the Native Hospital; and, with the concurrence of his Excellency the most noble Patron, and the Governors of that humane institution, formed a plan for extending its benefits to all who might desire it in Calcutta, as well as for securing a depôt of genuine matter under his own eye. He had also an opportunity of making experiments, in order to ascertain the efficacy of the cow-pock as a preventive of the small-pox.

The Medical Board at Calcutta, wisely considering that the preservation of the vaccine virus to Bengal, and perhaps to India, was a matter of too much importance to be trusted to the casual zeal of a few individuals, which might evaporate when the novelty of the practice was over, laid before the government a plan of an establishment every way calculated to secure to that country all the advantages of vaccination. This plan the Governor General ordered to be carried into effect. Subordinate superintendants of vaccination were appointed at eight different stations, in order to preserve and distribute the virus; under each of whom a number of the surgeons were directed to officiate, in promoting the general view of the institution. Thus the vaccine department was placed on a regular and permanent footing.

The Medical Officers on the establishment of the Company have in general shewn a disposition to co-operate with the superintendants, and done all in their power to promote the benevolent views of the government. But we are informed by Mr. Shoolbred, that the whole tribe of Bramin inoculators are, from interested motives, determined enemies of the new practice; and that, by their influence over the minds of the people, they have in many instances prevented them from bringing forward their children. This, together with the indolence and stupidity of the natives, will for a while prevent them from reaping the full benefit of so great a blessing.

After giving a detail of what has been done towards the propagation of the practice, Mr. Shoolbred takes notice of what still remains to be done. There are two places to which the Governor General is desirous of transmitting the matter; one is Port Jackson, the other China. To these two distant quarters it was proposed to send it in living subjects, by means of successive inoculations; but it was at that time impossible to procure a sufficient number of subjects for the purpose.

The number vaccinated in Bengal within the first year is upwards of eleven thousand. This number is not great; but the Bramins, the ancient inoculators, like too many of our ancient inoculators, have thrown obstacles in the way of the practice. Their sacred function gives them the greater authority; and the people themselves are ignorant, and of course prejudiced against all innovations.

By the publications of Dr. Anderson and Dr. Keir, we were taught to indulge a flattering hope, that the circumstance of this boon coming from the cow, an animal so highly revered by the Hindoos, would have operated in its favor; but we are informed by Mr. Shoolbred, it is directly the reverse.

After stating, that in Bengal one in sixty or seventy dies under inoculation of the small-pox, and one in three by the natural disease, and calculating the number of lives already saved, Mr. Shoolbred concludes his Report in the following manner:

" These are positive advantages already obtained by the introduction of vaccine inoculation into Bengal, which the intelligent part of society know how to estimate. What we have to look to in future appears to me to be, not only the continuance of the blessings before enumerated, but the the animating prospect of a sure and solid foundation having been laid for its universal diffusion in India, as well as to our eastern settlements, to Java, China, New South Wales, and even to the numerous islands scattered throughout the Pacific Ocean. In contemplating a period so auspicious to the happiness of mankind, and so glorious to the name of Jenner, we cannot help anticipating, that, while an approving Sovereign and an admiring Country do justice to the talents, the wisdom and energy so eminently displayed in the military and political career of our most noble and illustrious Governor General, it will not escape the notice of the philosopher and the philanthropist, that the same distinguished administration was no less conspicuous for the humanity, than for the vigour of its measures; and that to the encouragement afforded to vaccine inoculation, by His Excellency the Most Noble Marquis Wellesley, so large a portion of the globe has been indebted for the enjoyment of the inestimable blessings derivable from the greatest discovery that ever was made by man for the benefit of his fellow-creatures.

" JOHN SHOOLBRED,

" Superintendant General of Vaccine Inoculation."

*Calcutta, March* 22, 1804.

To this report is added an Appendix, containing practical remarks concerning the disease, as it appeared in India. It was introduced into Bengal in November 1802; and, in January 1803, Mr. Shoolbred put it to the test of variolous inoculation, which it resisted. It was also put to similar tests, as well as to that of exposure to the natural infection, by other gentlemen in different parts of India, with complete success.

Mr. Shoolbred would have repeated his experiments the following season, but the humane and judicious prohibition of variolous inoculation, in the neighbourhood of Calcutta, had cut off the supply of matter. This, however, he observes, is of little consequence, since the medical practitioners in that quarter of the globe had every reason to conclude, from the uniform and invariable character displayed by the disease, that the vaccine virus had suffered no diminution of power in their hands. On the contrary, from upwards of one thousand five hundred cases under his own care, as well as from the concurring evidence of all the subordinate stations, he was perfectly convinced, that the disease he then inoculated possesses in full force the prophylactic quality, which renders its discovery so inestimable a blessing to mankind.

"Even in the dark skin of the native of Bengal, the traits of genuine vaccination are sufficiently conspicuous to remove all doubts on the subject. The commencement of vesication on the fourth or fifth day, its gradual increase, circular form, depressed centre, cellular structure, and limpid contents; the surrounding tumefaction, or areola, where the skin is fair enough to shew it; the slight fever on the eighth, ninth, or tenth day, and the subsequent progressive conversion of the vesicle into its peculiar horny, dark-brown, glossy scab, dropping off from the fourteenth to the twentieth day, and leaving a permanent pitted cicatrix, are circumstances belonging to no other affection to which the human body is subject; and which, in my opinion, would stamp vaccination with the full evidence of its specific power, if no opportunity of putting it to the test of experiment ever occurred again."

As to the inoculation of the small-pox, Mr. Shoolbred says, it is truly believed, that although it often saved the individual, it has not lessened in the aggregate, the dreadful expenditure of human life occasioned by this disease.

He has not observed any other degeneracy of the virus from the high temperature and moist atmosphere of Bengal, than that it more frequently fails to communicate infection under those extremes. When it succeeds, the affection still exhibits all the essential characters of genuine vaccination.

Mr. Shoolbred copies from one of the Reports of the Vaccine Pock Institution, a passage containing an hypothesis of Dr. Pearson, in which he endeavours to explain the reason, why the virus becomes less efficacious after a certain period.

He supposes, that part of the active matter first secreted is absorbed; in consequence of which, a change is effected in the constitution, which puts a stop to the secretion of vaccine matter; and the secretion then becomes of a serous kind.

It is certain, however, from the success of vaccination with matter from the dry scab, that a great portion, if not the whole of the active particles, remain unabsorbed. I therefore beg leave to submit with deference another hypothesis, by way of solving this problem; namely, that simple dilution, independent of absorption, may occasion the temporary inefficacy of the matter; and simple inspissation, sation, in consequence of evaporation by the cuticle, may restore it to its pristine activity.

Mr. Shoolbred made some experiments, in order to determine how far Dr. Jenner's opinion was well founded, that persons who have had the small-pox, or the cow-pock, are still susceptible of the latter. Mr. Shoolbred is correct in his idea, that such are much less susceptible of the disorder than before. This, however, is the very doctrine originally taught by Dr. Jenner, and confirmed by other authorities, whatever has been insinuated to the contrary by any illiberal opponent. Several instances of the cow-pock taking place a second time, are to be found in the writings of Dr. Jenner; and, in the index of my Treatise on the cow-pox, I have referred to no less than ten places, where instances are adduced of its occurring after the small-pox. Any one who takes the trouble to examine those cases will be able to judge, how far the persons on whose authority they rest, merit the title given them by Dr. Pearson,—that of a few partizans.

Mr. Shoolbred candidly admits, that from my Treatise it appears, some persons have had the cow-pock after the small-pox. We are therefore, he observes, not warranted in saying that such a thing never happens; but only that it happens too seldom, to afford any reasonable prospect of keeping up the virus this way.

From the same source Mr. Shoolbred learned, that cows had been vaccinated from the human subject; and that the disease had been restored to the human subject again. He informs us, however, that before my treatise fell into his hands, he had made the same experiments; principally with a view of ascertaining the appearance of the complaint in the cow.

One striking difference which Mr. Shoolbred discovered between the cow-pock in the cow and in the human subject, was, that in the former, instead of being vesicular, it had more the appearance of an elevation of the substance of the teat itself. The vaccine fluid was limpid, but only to be obtained by pushing a lancet under the scab.

The only sign of constitutional affection was a diminution of milk; but whether this was owing to a discontinuance of milking, Mr. Shoolbred is unable to determine. The tumours resembled those represented by Dr. Sacco, or those which take place on applying a thimble exhausted of air on any fleshy part of the body. These experiments were instituted with a view to facilitate the preservation of cow-pock matter. The mode he prefers, is that of

 a cona continual succession of inoculations in the human subject.

Mr. Shoolbred has made two very interesting inquiries; the first is, whether the indigenous cow-pox has ever been discovered among the cattle in India; the second, whether the Bramins were acquainted with the practice of vaccination before the promulgation of Dr. Jenner's discovery, as some people have lately alleged.

From the result of his inquiries, there is great reason to doubt whether the indigenous cow-pock has ever appeared in India; and the pretence of the Bramins to a previous knowledge of vaccination, is proved to be one of the basest forgeries ever attempted.

A Shanscrit manuscript was handed about, which, it was pretended, contained an account of the practice; but it was remarked, that the operation was directed to be performed after the European, and not the Asiatic manner.

The manuscript in question was therefore compared with other copies, and the imposition was detected. The part relative to the cow-pock was an interpolation; or, to speak in the emphatic language of Mr. Shoolbred, an impudent forgery. Such interpolations, I learn from this and other sources, are commonly practised in India, for the purpose of supporting the claims of the Bramins to priority in every kind of science.

They only practise inoculation of the small-pox during three months in the year; and then only in a partial manner; for we are told by Mr. Shoolbred, it is almost entirely confined to Bengal. Had the Bramins practised vaccination, they would not now be among the first to oppose it. The grease, it appears, is not known in India.

The small-pox has not prevailed epidemically in Calcutta since vaccination was introduced there, owing to the prohibition of variolous inoculation. A few sporadic cases have, however, occurred, sufficient to furnish matter for counter-proofs.

Mr. Shoolbred doubts, whether the natives have sufficient capacity to practise vaccination with success; but he has no doubt that they possess sufficient knavery to contaminate the matter, in order to bring it into discredit. He says, no sensible and conscientious man in Great Britain thinks of continuing variolous inoculation, since the value of vaccination is fully ascertained. The artificial introduction of the small-pox into Calcutta, has annually consigned thousands of victims to an untimely grave; and it is supposed, that scourge of humanity would have have been less fatal, had it been left to pursue its natural course.

In your Journal for November last I mentioned, that Dr. Anderson of Madras, proposed to send a surgeon with cow-pock matter, and some children who are proper subjects for vaccination, to New South Wales, for the sake of conveying the virus in a state of activity to that remote region. In a former part of the present communication I observed, that the Governor General of India was also desirous of carrying this plan into execution; but that it was impossible to procure a sufficient number of subjects for the voyage. It is, therefore, with peculiar satisfaction, that I have just received advices from Mr. Savage, of the accomplishment of this important object, by means of vaccine virus which he received from me.

This event is the more memorable, on account of the length of the voyage, and the heat to which the matter must have been exposed in crossing the line. It has, indeed, been generally concluded, that under such circumstances, it was impossible it should ever prove successful. Our countrymen in India seem to have considered the conveyance of it from that quarter as a hopeless attempt, unless it were conveyed by successive inoculations, and a ship-load of children.

It has long been thought impracticable to transmit active matter to India by sea, though a shorter voyage. The failure of all the attempts hitherto made has been attributed to the effect of time, or heat, or the effluvia of various kinds to which matter is exposed during the passage. The last cause I have always considered imaginary, from the mode in which the virus has been packed up. The only cause to which I can ascribe the universal failure of such attempts, and of Mr. Savage's first matter which he carried out with him is, the difference of the vehicles.

The former were thread and glass. The first of these is nearly the same with the vaccinator, since it admits of being applied without diluting the matter. Glass is incapable of that advantage; and is more apt to fail than any other method.

Having in a great measure supplied the army with matter for almost two years, I have had ample opportunities of verifying this fact.

On a former occasion I noticed, that some of the natives of New South Wales bear evident signs of the small-pox. I have now before me a concise history of the English colony in that quarter; from which I shall extract a paragraph, graph, that renders it probable the disease was first introduced into this part of the world, since the settlement was established.

"When that shocking and fatal malady, the small-pox, first made its appearance amongst these unfortunate creatures, says Governor Hunter, it was truly shocking to go round the coves of the rocks, where nothing was now to be seen, but men, women, and children, lying dead. As we had never seen any of these people, who had been in the smallest degree marked with the small-pox, we had reason to suppose they had never before been affected by it, and consequently are strangers to any method of treating it; and if we consider the different attitudes the dead bodies have been found in, we may easily believe, that when the malady assumes the appearance of this disorder, they are immediately deserted by all their friends, and left to perish for want of sustenance."

Mr. Savage, in his last letter, informs me, that when the small-pox was introduced into Botany Bay, among the natives, many hundreds fell victims to this merciless disease; and that, on numbers of those miserable wretches, it has left its ghastly traces behind.

He adds, "Nothing can appear finer than the pustule in every instance; and the disease, if such it can be called, observes precisely the form described by Dr. Jenner and yourself. A little drowsiness on the fourth, but more generally on the fifth day, is the only symptom; except, in one instance, a native boy of New Holland, servant of the Rev. Mr. Marsden, who complained much of head-ach, and some feverish symptoms.

"Your zeal in the cause of humanity will be highly gratified at our joint success, in this fifth grand division of the globe; and our Friend Harwood, who rejoices 'in all the good of all mankind,' and who was in some measure instrumental to the introduction of the vaccine-pock into this country, will derive equal satisfaction from the event.

The following articles appear in the Sydney Gazette of the 3d of June.

"Notice.

"As the cow-pox is now fully established in the colony, it is hoped no parents or guardians of any children will omit availing themselves of so great a blessing; which, as has been shewn in the Gazette of the 13th of May last, is an infallible preventive of that generally fatal distemper, the small-pox.

"Notice.

"NOTICE.

"Mr. Harris, Surgeon of the New South Wales Corps, informs the parents of children belonging to the military, that he will inoculate with the vaccine infection, those who choose to attend him, at his house at Sydney, every Tuesday, and at his house at Parramatta, every Saturday, till further notice."

In the aforesaid Gazette is a letter from Mr. Jamison and Mr. Savage to the Governor, in which they inform him, that vaccine matter from another source failed; but the matter received from me succeeded; and Mr. Savage having vaccinated several children from that in which infection first took place, they are decidedly of opinion that the cow-pock is completely established in the colony. To further its propagation, they request that the Governor will give orders for parents to attend at the hospital at Paramatta. They promise to give notice, when it is established at the other settlements.

The following General Orders are inserted in the same Gazette:

"Such children as the parents or guardians may wish to have inoculated, are to attend at Parramatta, on Tuesday and Wednesday next; after which a permanent attendance will be directed at Sydney, Parramatta, and Hawkesbury.

"By Command of His Excellency,
"G. BLAXCELL, Acting Secretary."

In his letter, dated August 3, 1804, Mr. Savage says: "A great number of subjects have now been inoculated; and, to prevent the possibility of losing the virus, we limit the numbers to four or five at a time, in each settlement."

Whenever this topic is introduced, it cannot be unseasonable to remind medical practitioners, that it is always proper for them to keep matter, taken at an early period, on vaccinators. From the foregoing account it is evident, that these, when well charged, even under the disadvantage of a warm atmosphere, will preserve matter in an active state many months.

I am, &c.

JOHN RING.

*New Street, Hanover Square, Feb. 15, 1805.*

